# Shading Affects Yield, Elemental Composition and Antioxidants of Perennial Wall Rocket Crops Grown from Spring to Summer in Southern Italy

**DOI:** 10.3390/plants9080933

**Published:** 2020-07-23

**Authors:** Gianluca Caruso, Luigi Formisano, Eugenio Cozzolino, Antonio Pannico, Christophe El-Nakhel, Youssef Rouphael, Alessio Tallarita, Vincenzo Cenvinzo, Stefania De Pascale

**Affiliations:** 1 Department of Agricultural Sciences, University of Naples Federico II, 80055 Portici, Naples, Italy; luigi.formisano@unina.it (L.F.); antonio.pannico@unina.it (A.P.); christophe.elnakhel@unina.it (C.E.-N.); youssef.rouphael@unina.it (Y.R.); lexvincentall@gmail.com (A.T.); vincenzo.cenvinzo2@unina.it (V.C.); 2 Council for Agricultural Research and Economics (CREA)-Research Center for Cereal and Industrial Crops, 81100 Caserta, Italy; eugenio.cozzolino@crea.gov.it

**Keywords:** *Diplotaxis tenuifolia* L. (D.C.), sustainable management, shading nets, cropping seasons, leaf production, minerals, phenols, ascorbic acid, antioxidant activity

## Abstract

Shading nets have been increasingly drawing research interest, as they allow us to improve the environmental conditions for greenhouse-grown crops. The effects of two shading nets (50% and 79% shading degree), plus an unshaded control, on yield, mineral composition and antioxidants of perennial wall rocket (*Diplotaxis tenuifolia* L.-D.C.) grown under tunnels in southern Italy were determined. The shading application resulted in a yield decrease, compared to the unshaded control, except for the highest production under 50% shading in July. The highest yield was recorded in the April–May and May–June and the lowest in July. Similar trends were recorded for plant dry weight, leaf number per rosette and mean weight, but the latter showed the highest value under 79% light extinction in July. The rocket leaves were brighter in the summer cycles than in the spring ones. Leaf nitrate was highest in spring and under 79% shading. Potassium, phosphorus, calcium and magnesium showed the highest values in spring and in the unshaded control. The lipophilic antioxidant activity showed the highest values under the 79% shading net in the spring cropping seasons, whereas in July it did not significantly differ from 50% light extinction. The hydrophilic antioxidant activity always attained the highest values in the unshaded control. The unshaded leaves had the highest total phenol accumulation when grown in April–May and the lowest in July. The total ascorbic acid content was always highest in the unshaded control leaves compared to the shading treatments. Fifty percent crop shading is, therefore, an effective sustainable tool for increasing the yield of perennial wall rocket leaves in July, when the light intensity under the plastic tunnel exceeds the plant requirements, also resulting in a mineral composition that is not significantly different from that of the unshaded crops.

## 1. Introduction

*Diplotaxis tenuifolia* L., commonly named perennial wall rocket, is spread worldwide, oriented both to the fresh salad market and the baby leaf industry [1], appreciated by consumers for its bitter flavor, and rich in beneficial phytonutrients such as vitamin C, glucosinolates and flavonoids [2].

Perennial wall rocket needs proper levels of light intensity and air temperature to encourage plant growth as well as leaf yield and phytochemical content [3,4,5]. In order to modulate the aforementioned environmental factors, different strategies can be adopted, among which are the use of shading nets, which can contribute to improving the plant growing conditions, thus leading to more vigorous plants, higher yields and better quality produce [6,7].

Shading nets are characterized by different mechanical, physical and optical properties [8], which allow for the modulation of light and temperature levels around crops. Interestingly, the shading nets can concurrently influence the quality and quantity of sunlight radiation, taking into account that some of them, such as the grey- or black-colored nets, do not alter the spectral composition of light, but just reduce its intensity [9,10,11]. The photoselective screens increase the diffused radiation, normalize excessive levels of light, temperature, humidity and wind velocity [12] which allow for the greater efficiency of vegetable production in protected cultivation [13]. In addition, photoselective nets improve the quality of vegetables at harvest [14] and at the post-harvest stage [15,16,17].

An experiment carried out by Jin et al. [18] showed the effects of light conditions on wild and salad rocket: compared to high light intensity (80–120 μmol·m^−2^·s^−1^), under low light intensity (20–30 μmol·m^−2^·s^−1^) the plants had larger leaf area, a 40% lower antioxidant content, and reduced levels of glucosinolate, quercetin, isorhamnethin, kaempferol, and cyanidin. Francke [19] reported, in *Diplotaxis tenuifolia* and *Eruca sativa*, a higher N and K accumulation under reduced light conditions, whereas P and Ca were higher in the unshaded control.

Recent research revealed that lettuce grown without shading had a lower content of flavonoids if compared with shade net treatments [20]. Otherwise, photoselective nets did not affect the glucosinolate content in turnips (*Brassica rapa* subsp. *rapa* L.), a parameter probably related to genotype and planting date [21].

Ombodi et al. [22] reported that shading nets led to 15–40% light extinction and caused significant losses in sweet pepper hybrid yields under a plastic tunnel: the production of Karpia F_1_ decreased from 8.5 to 6.0 kg·m^−2^, that of Karpex F_1_ decreased from 7 to 6 kg·m^−2^. Other authors showed that organic pepper benefited from the application of shading nets with light extinctions of 25% and 35%, compared to the unshaded control [23].

Rocket is one of the few C_3_–C_4_ *Brassicaceae* species [24], and better assimilates CO_2_ at irradiance levels of 600–900 μmol·m^−2^·s^−1^, i.e., about 30–40% of the sunlight radiation commonly recorded in the late spring–summer growing season in Mediterranean areas [20]. Indeed, when the radiance energy exceeds the optimal genotype threshold for net photosynthetic assimilation, photo-inhibition is activated along with stress reactions, such as stomatal closure, cell division, leaf expansion and reproductive development [25]. Contrarily, the low irradiation level elicits changes in plant morphological and chemical features, leading to broader and thinner leaves, a less dense canopy and phytochemical content modulation. In the latter respect, the light and temperature inside the greenhouse should encourage the leaves of perennial wall rocket to achieve an appropriate shape, with petioles not excessively long in comparison with the blades, and an appreciable concentration of phytochemicals [26,27]. The aforementioned environmental parameters also affect the crop performance depending on the cropping season, which, in a previous work, influenced the dry matter and macronutrient content as well as the vitamin C, phenols and total glucosinolate concentration in leaves of soilless-grown rocket [26].

The use of shading nets is one of the strategies aimed at protecting plants from exceeding values of radiation and temperature during the spring–summer crop cycles of *Diplotaxis tenuifolia* L. In the latter respect, the purpose of this research was to investigate the effect of two shading nets characterized by different light extinction levels (50% and 79%, plus an unshaded control) on the yield, mineral composition and antioxidants of perennial wall rocket oriented towards the fresh market, grown in a greenhouse in four different spring–summer cycles in southern Italy.

## 2. Results and Discussion

### 2.1. Meteorological Parameters

**Figure 1:**
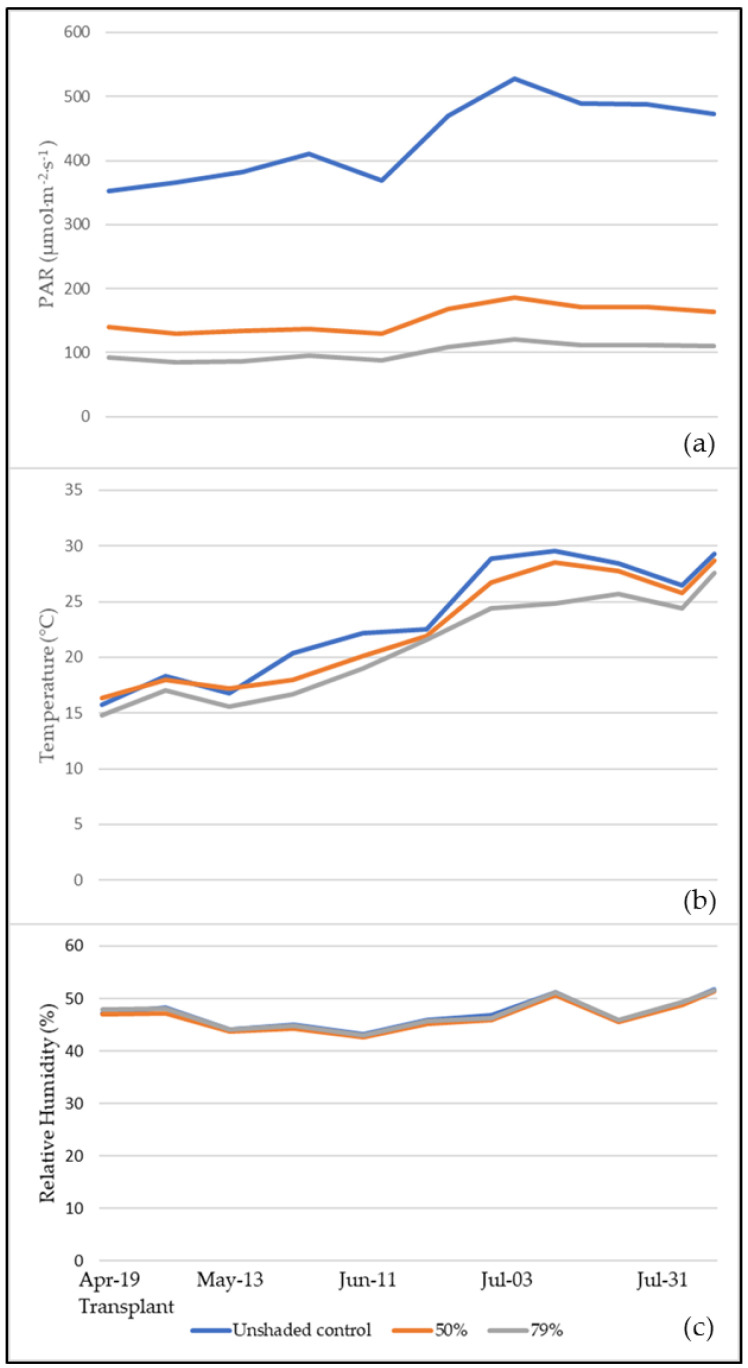
Seasonal trends corresponding to 50% and 79% shading nets and unshaded control under tunnels of: (**a**) photosynthetic active radiation (PAR); (**b**) temperature; (**c**) relative humidity. The dates reported on the x axis correspond to the end of each cropping season in the unshaded control, from the first (13 May) to the fourth (31 July).

The trends of mean Photosynthetic active radiation (PAR), temperature and humidity in the greenhouse are shown in Figure 1a–c. The PAR generally exceeded the 400 µmol·m^−2^·s^−1^ level and sometimes even 500 µmol·m^−2^·s^−1^ (with 527 µmol·m^−2^·s^−1^ as a maximum value) in the June–July and July cropping seasons in the unshaded control, which was 2.84-fold and 4.32-fold higher on average compared to 50% and 79% shading, respectively. In April–May and May–June crop cycles, the mean PAR ranged between 350 and 410 µmol·m^−2^·s^−1^ in the unshaded control, whereas it showed a 64.6% and 76.8% decrease corresponding to the 50% and 79% light extinction rates, respectively.

The mean daily temperature increased from the transplant to the beginning of the last cropping season in July: in the unshaded plots, it was 5.93% and 15.01% higher compared to 50% and 79% shading respectively. The reduction in PAR values obtained in the current experiment are consistent with previous studies [28,29,30].

Unlike the trends of PAR and temperature, humidity values inside the greenhouse remained steady and did not vary between the crops under different shading nets.

### 2.2. Plant Growth and Yield

The main effects of the two experimental factors applied in the present research are shown in Table 1. The crop cycle was longest in May–June and under 79% shading, and shortest in July; the yield variables examined generally showed a decreasing trend both from the first cropping season to the fourth, and from the unshaded control to 79% shading.

**Figure 2:**
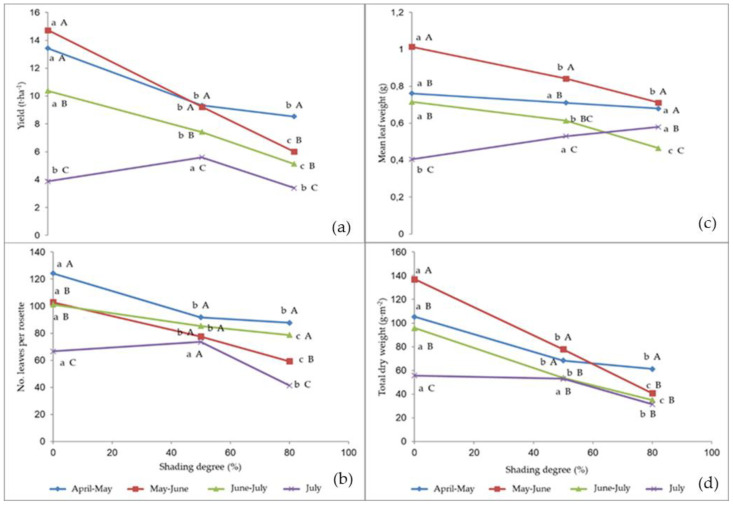
Interaction between cropping season and shading degree on: (**a**) leaf yield; (**b**) leaf number per rosette; (**c**) mean leaf weight; (**d**) total dry weight. Values followed by different letters are significantly different according to the Duncan test at *p* ≤ 0.05. Lowercase letters refer to the comparison between the shading treatments within each cropping season, and capital letters refer to the comparison between cropping seasons within each shading treatment.

The interaction between the cropping season and the shading degree was significant on the yield parameters and plant dry matter (Figure 2a–d).

The shading application resulted in a yield decrease, compared to the unshaded control, over the first three cycles from April to the end of June, whereas the crops grown in July showed a production increase under 50% shading (Figure 2a); 79% light extinction always caused the worst performance, but in April–May it did not significantly differ from the May–June cropping season. The highest yield of the unshaded control was recorded in the April–May and May–June crop cycles, and the lowest in July; 50% shading led to the highest yield production in April–May and May–June crop cycle, followed by June–July and July crop cycle respectively; the yield corresponding to 79% light extinction was highest in April–May and lowest in July.

The number of leaves per rosette (Figure 2b) decreased both with increasing the shading and when delaying the crop season, except for the July cycle, when the leaf number did not significantly change from the unshaded control up to 50% shading. In the unshaded control, the highest leaf number was recorded in the April–May crops, and the lowest in the July ones, with no differences between the intermediate cycles. Under 79% shading, the cycles April–May and June–July showed the highest number of leaves. No differences arose between the cropping seasons at 50% shading. The highest differences between 50% and 79% shading were recorded in the July cycle.

The mean leaf weight (Figure 2c) did not show significant differences between the shading treatments and the unshaded control in the April–May cycle; it was highest in the unshaded control in May–June and June–July; in the July cycle, shading led to higher mean leaf weight compared to the control. Both in the unshaded control and under the shading treatments, the leaves harvested in May–June attained the highest mean weight, though the latter was not significantly different from that recorded in April-June regarding 79% light extinction.

The highest dry weight content (Figure 2d) was recorded in the unshaded rocket leaves in all the cropping seasons, except for July when it was not significantly different from that associated to 50% shading. The control resulted in the highest dry weight content in May–June, 50% shading in April–May and May–June, and 79% light extinction in the April–May cycle.

In the present investigation, increasing shading caused a decreased yield, except for the crop cycle in July, characterized by the highest light intensity (Figure 1a), which was better affected by 50% shading compared to the unshaded control. Presumably, the light intensity recorded in July exceeded the perennial wall rocket light requirements and, therefore, the crops benefited from a 50% light reduction. Indeed, the excessive irradiation, over 600–900 μmol·m^−2^·s^−1^, elicits a leaf temperature increase in C_3_ plants, leading to a photoinhibition effect [31] as well as imbalances in rubisco activity [32], electron transport [33], and stomatal and mesophyll conductance [34]. In this respect, Santamaria et al. [35] recorded a 50% increase in dry matter in rocket plants with a light intensity reduction from 20 to 10 klux.

As reported by Padulosi and Pignone [36], rocket is a cool-season crop that shows a shorter cycle with an increase in day length and temperature, consistently with what was recorded in our study. However, the 79% shading level applied in the present research always caused a dramatic reduction in the sunlight radiation entering the greenhouse, whose intensity proved to be under the optimal light needs of *D. tenuifolia* plants. In a previous study, based on a comparison among different leafy vegetable species, Wolff and Coltman [37] highlighted that the crops positively benefited from shading up to 30%–47%, with lettuce showing a 36% yield increase and head and Chinese cabbage a 23% and 21% augmentation, respectively. Kavga et al. [38] reported that, in a comparison between lettuce and rocket crops grown under a 25% shading net, only the rocket crop showed a yield loss up to 50% compared to the unshaded control. In contrast, Ilić et al. [30] recorded an increased yield of *Lactuca sativa* L. grown under different 50% shading nets; specifically, the leaf area index, the total fresh weight, the leaf number per plant and stem length increased under shading in comparison with the unshaded control, suggesting the existence of a light-dependent mechanism by which the plants regulate the leaf size.

In another study carried out by Caruso et al. [23] on organic pepper, 25% and 35% light extinction resulted in yield increases of 13.5% and 8.1%, respectively, compared to the unshaded control, as a consequence of the 19.4% and 11.3% enhancements of the fruit numbers per plant.

### 2.3. Leaf Color Parameters and Chemical Composition

In the present study, the rocket leaves were brighter in the summer cycles than in the spring ones, as reflected by the higher L* values shown in Table 2. No significant differences arose between the shading nets and the control.

The a* and b* color components were not significantly affected by either the cropping season or the shading net. Our findings are in agreement with the results achieved by Ilić et al. [30] in a previous study aimed at comparing the performance of photoselective shading nets on the visual quality attributes of lettuce in a summer cycle.

As no significant differences arose between the April–May and May–June cropping seasons regarding the macro- and microelement contents as well as the antioxidant compounds and activity, only the results relevant to the April–May crops have been reported in the Table 3, Table 4 and Table 5.

Among the macronutrients analyzed (Table 3), total nitrogen, sulfur and sodium in perennial wall rocket leaves were not affected by either the cropping season or the shading degree. On the other hand, nitrate showed decreasing values from spring to summer seasons, but increasing concentrations with crop shading enhancements. The potassium content was higher in the leaves grown in summer compared to the spring ones and was increasingly inhibited from the unshaded control to 79% light extinction. Phosphorus, calcium and magnesium showed the highest values in the spring cropping season and without shading.

With regard to the micronutrients in *D. tenuifolia* leaves (Table 4), the contents of Cu, Fe and Se increased from April–May to July, whereas Zn showed the opposite trend, and Mn was not significantly affected by the cropping season. Moreover, the levels of all micronutrients were highest in the unshaded conditions and lowest under 79% shading, except for Se, which accumulated the most in the 50% shaded leaves.

In agreement with the study of Tindall et al. [39], who found that 25 °C was the suitable temperature for the optimal mineral uptake, in the present investigation, the moderate temperatures recorded in April and May led to higher mineral contents compared to summer cropping seasons, except for K. Gregory [40] also reported the increase in NO_3_, Ca, P and Mg at temperatures ranging between 20 and 30 °C, and, in this respect, the plant mineral uptake is affected by the soil temperature, which elicits changes in the root physiology and architecture. Moreover, the air temperature influences the growing relationships between shoots and roots and, accordingly, the photosynthate translocation pattern [34].

In contrast to our results, which are relevant to perennial wall rocket leaves, Stagnari et al. [41] found, in a greenhouse-grown lettuce, rising trends in mineral contents from the unshaded control to 85% PAR reduction, by 1.18-fold for Ca, 1.26 for P, 1.67 for Mg, and 2.89 for K. Díaz-Pérez [42] recorded an increasing content of N, P, K and Na with a shading degree increase from 0 to 80% in bell peppers, but the other elements’ content decreased. Zhao and Oosterhuis [43] also showed the benefit of shading on leaf mineral content: cotton plants grown under 63% light reduction accumulated much more minerals in the leaves, especially N, P, and S, in comparison with the unshaded control. In a study carried out by Chen et al. [44], the fruit content of N, P, K, and Mg increased under 60% shading, whereas the Ca level decreased. In a further study, Stagnari et al. [45] reported contrasting effects of a green shading net on red turnips, resulting in a lower dry weight of roots and leaves, but an increase in the concentration of soluble and structural carbohydrates, as well as of K, Mg and Zn.

*D. tenuifolia* has a physiological tendency to accumulate nitrate, which is a potential health risk to consumers at high concentrations [46], and therefore related recommendations are reported in the European Union Regulation N. 1258/2011. However, Steinmetz and Potter [47] reported that high antioxidant contents can inhibit the formation of carcinogenic compounds. In agreement with the results of the present research, the accumulation of NO_3_ in plant tissues was enhanced under reduced light intensity in previous investigations on rocket [46] and spinach [48]. In fact, nitrate reduction to nitrite and the ultimate conversion into organic compounds is achieved by the nitrate reductase enzyme complex, whose synthesis, induction and reducing power through photosynthesis is positively correlated with sunlight intensity [49].

### 2.4. Antioxidant Compounds and Activity

In this study, the interactions between the cropping season and the shading degree were significant both on antioxidant activity and compounds (Table 5). Indeed, lipophilic antioxidant activity (LAA) showed the highest values under the 79% shading net in the first and second cropping seasons, whereas in the July cycle it was not significantly different when compared to 50% light extinction; the unshaded leaves had the lowest LAA, except for the first cropping season, which did not differ from the second one.

The hydrophilic antioxidant activity (HAA) always attained the highest values in the unshaded control, but in April–May it was not significantly different from that recorded in June–July; the lowest HAA was detected in the last cropping season.

The results of the present research are consistent with those obtained by Colonna et al. [50], who found that LAA was higher under low PAR (200–400 µmol·m^−2^·s^−1^) compared to high PAR (800–1200 µmol·m^−2^·s^−1^) conditions. Contrastingly, Jin et al. [18] reported that the full light conditions caused the increase in rocket antioxidant activity compared to the lower light intensity. Indeed, light intensity is stressful to plants when its values are either above or below the optimal threshold related to the specific crop system requirements. In this respect, in the present investigation, LAA showed the highest levels both in July when the sunlight exceeded the perennial wall rocket demands and under the 79% shading, which caused an excessive PAR reduction inside the tunnels.

The total phenols did not show unequivocal trends as a function of the cropping season or the shading degree. Indeed, the unshaded leaves had the highest accumulation of these antioxidants when grown in April–May and the lowest in the July cropping season; controversial trends in the three cropping seasons were related to the two shading degrees.

The total ascorbic acid (TAA) content was always highest in the unshaded control; within each of the shading treatments, the highest values were recorded in July in the unshaded leaves, in April–May in the 50% shaded ones and in June–July in those grown under 79% light extinction.

Many factors affect the antioxidant content, such as air temperature, harvesting time [51] and light. In the latter respect, Luthria et al. [52] found that the UV radiation range between 290 and 400 nm better influences the phenolic acid concentration in tomato fruits than the 380–400 nm range. Indeed, optimal light conditions and UV, in particular, are reportedly essential for optimizing the concentration of phenolic compounds, partly because phenolic compounds have a strong capacity for UV radiation absorption [53].

In a study carried out by Cano and Arnao [54], the lipophilic antioxidant activity of lettuce leaves was directly related to the efficiency of photosynthesis; in fact, the youngest leaves showed a lower LAA value. In the present study, the LAA was higher in the summer crop leaves, which were subjected to the most intensive sunlight radiation and in leaves under the 79% shade level, maybe due to the fact that the photosynthetic activity was distributed among fewer leaves.

Polyphenols constitute a heterogeneous natural substance, whose accumulation in plants is reportedly genotype dependent [55], noted for their beneficial effects on human health. Recent studies have shown the positive effect of phenolic compounds in constraining carcinogenic cell development [56,57]. Furthermore, their strong antioxidant capacity reduces the side effects associated with various diseases of the nervous system [58,59,60]. The phenol content recorded in the present experiment under the shading net treatments is comparable with the results of Ilić and Fallik [20]. During a summer cycle of *Lactuca sativa* L., photoselective shading nets did not improve the total phenol content compared to the unshaded control. Oh et al. [61] reported the negative effect of the 40 to 50% PAR reduction on the accumulation of phenolic compounds, in contrast with a similar experiment in which the plants were subjected to a high light intensity [62,63]. In previous research carried out by Wang et al. [64], the total phenolic and flavonoid contents were significantly affected by the shading application.

Ascorbic acid is a major vitamin and antioxidant in vegetables [65], and in the present investigation its content was higher in the control than in the shaded rocket leaves, confirming the positive correlation of this compound with the light intensity, consistently with the reports of Kosma et al. [66]. The latter authors reported that the 27% shading in greenhouse-grown lettuce elicited the highest leaf ascorbic acid content, because it presumably enhanced the plant’s photosynthetic performance compared to 53% and 74% shading. Indeed, the synthesis of ascorbic acid is encouraged under the optimal light conditions relevant to the specific crop and growing season, as previously reported in tomatoes [67], and in *Arabidopsis thaliana*, where a positive correlation between the ascorbic acid accumulation and the light intensity was found in plants grown under 50-µmol photons m^−2^ s^−1^ and 250-µmol photons m^−2^ s^−1^ light intensity [68].

In a previous study [23], the ascorbic acid content in pepper fruits was positively correlated with the shading degree, increasing by 31.1% from the unshaded control to 35% shading treatments.

## 3. Materials and Methods

### 3.1. Growing Conditions and Experimental Protocol

Research on the perennial wall rocket (*Diplotaxis tenuifolia* (L.) D.C.) cultivar Nature was carried out in the experimental fields of the Department of Agricultural Sciences of Naples University Federico II in Portici (Naples, southern Italy, 40°49′ N, 14°15′ E, 72 m a.s.l.) in 2019. The trial was conducted under three tunnels, each of them 5.0-m wide, 30-m long, 2.0- and 3.5-m tall at wall and roof, respectively, covered with a thermal polyethylene film, in a sandy loam soil (76% sand, 17% silt, 7% clay), with a pH of 6.9 and an electrical conductivity of 512 mS cm^−1^, from 19 April to 31 July.

In each crop cycle, continuous measurements of PAR, air temperature and relative humidity were performed, both under shading nets and in an unshaded control. In addition, periodic measurements of PAR were taken four times during the daily light period between sunrise and sunset in order to check the net shading degree.

The rocket rosettes were arranged in four rows per bed, mulched with a biodegradable film, with a 20-cm spacing both along and between the rows, with a density of 14.3 rosettes per m^2^.

The experimental protocol was based on the comparison between two shading nets (Frangisole 50, 50% light extinction, and Frangisole Iron 90, 79% light extinction; both nets were provided by Arrigoni S.p.A, Uggiate Trevano, Italy) plus an unshaded control, each of them corresponding to a tunnel, in factorial combination with four crop cycles (April–May; May–June; June–July; July). A randomized complete block design was used with three replications, and the experimental unit had a 6.4-m^2^ surface area.

The first crop cycle began on 19 April and ended on 13 May, 15 May and 20 May in the unshaded control, 50% and 79% shading treatments, respectively. The second crop cycle ended on 11 June in the control, on 13 and 17 June in the plots under 50% and 79% shading, respectively. The third cycle lasted until July 3 in correspondence of the unshaded control, 4 and 8 July corresponding to 50% and 79% shading respectively. The fourth crop cycle ended on 24, 26 and 31 July in the control, 50% and 79% shaded treatments, respectively.

The perennial wall rocket crops were managed through the following sustainable farming practices: organic fertilization prior to transplant with N, P_2_O_5_ and K_2_O (at a rate of 38, 10 and 30 kg·ha^−1^, respectively); 15-µm-thick MaterBi biodegradable black mulching; protection against fungal diseases and pests with copper oxychloride and azadirachtin treatments, respectively; drip irrigation when the soil available water at 10 cm depth dropped to 80%, based on the crop evapotranspiration [69]; N, P_2_O_5_ and K_2_O supply by fertigation at a dose of 112, 30 and 90 kg·ha^−1^, respectively.

At harvest, the rocket leaves at the marketable stage were cut to 12- to 15-cm lengths, at 3 to 5 cm above the soil surface, so as to safeguard the vegetative apex and allow for a more efficient re-growth [27]

At each harvest time, on random samples taken in all the experimental plots, yield and colorimetric determinations were performed, while mineral composition and antioxidant compound activity were measured in the laboratory.

### 3.2. Dry Weight

The assessment of leaf dry weight was done after the dehydration of the fresh samples, at 70 °C until a constant weight was reached, in a forced-air oven.

### 3.3. Leaf Colorimetric Parameters

The leaf color parameters L*, a* and b* were measured on the central area of the upper surface of 10 leaves per replicate by means of a Minolta CR-300 Chroma Meter (Minolta Camera Co. Ltd., Osaka, Japan) [27].

### 3.4. Mineral Elements

The content of, P, K, Na, Ca, NO_3_-N, Mg and S was measured in leaf dry tissues ground in a Wiley Mill and then sieved through an 841-micron mesh. To prepare the samples, 250 mg of leaf tissue powder suspended in ultrapure water (50 mL) (Milli-Q, Merck Millipore, Darmstadt, Germany) underwent three freeze-thaw cycles in liquid nitrogen and was then shaken in a water bath (ShakeTemp SW22, Julabo, Seelbach, Germany) at 80 °C for 10 min. The resulting mixture was managed according to the procedure of Rouphael et al. [70] and the determinations of the mineral elements were performed in compliance with the same method [70].

For the determination of the total nitrogen concentration, the Kjeldahl method as described by Bremner [71] was used, and the results were expressed as the percentage of N in the plant sample.

### 3.5. Antioxidant Compounds and Activity

The total phenolic content in methanolic extracts was assessed using the Folin–Ciocalteu method with gallic acid as a standard. Five hundred mg of freeze-dried material was extracted in 60% methanol (10 mL), placed on a shaker for 15 min and then centrifuged for 5 min 4000× *g*. One hundred µL of the supernatant was combined with 500 µL of Folin–Ciocalteau’s reagent (Sigma-Aldrich Inc., Milano, Italy) and 400 µL of 7.5% sodium carbonate/water (w/v). After 30 min of incubation in the dark at room temperature, the solution absorbance was measured at 765 nm by an ultraviolet–visible spectrophotometer, expressing the results as mg gallic acid (Sigma-Aldrich Inc.) per 100 g of dry weight.

The total ascorbic acid (TAA) was determined by a spectrophotometric method as described by Kampfenkel et al. [72], by reducing the dehydroascorbate to ascorbic acid upon the sample preincubation with dithiothreitol. The solution absorbance was measured at 525 nm, expressing the results as mg ascorbic acid per 100 g fresh weight.

Lipophilic antioxidant activity (LAA) was determined according to Re et al. [73], and the hydrophilic antioxidant activity (HAA) in compliance with Fogliano et al. [74].

### 3.6. Statistical Processing

The data were analyzed by the two-way analysis of variance using the SPSS software version 21, and the Duncan multiple range test was performed for mean separations at a 0.05 probability level. The data, expressed as percentages, were subjected to angular transformation before processing.

## 4. Conclusions

From a study carried out in southern Italy on perennial wall rocket (*Diplotaxis tenuifolia* L.-D.C.) grown under tunnels, it arose that the unshaded crops showed the highest yield from mid-April to late June, whereas the application of a 50% shading net produced a beneficial effect on the leaf production in July, in relation with the highest PAR and temperature values, whereas the 79% shading proved to limit the plant light requirements in any cropping season. The unshaded crops generally showed higher mineral accumulation compared to the 79% light extinction, but the mineral accumulation was not significantly different from that elicited by the 50% shading. Interestingly, the latter treatment resulted in the highest content of selenium, a microelement acting as an effective antioxidant. The ascorbic acid and the connected hydrophilic antioxidant activity were best affected by the highest light intensity, whereas the opposite trend was shown by the phenols and lipophilic antioxidant activity. The shading nets proved to be an interesting tool within sustainable horticultural systems, though the optimal degree of light extinction for achieving the highest yield and produce quality depends on the cropping season.

## Figures and Tables

**Table 1:** Rocket yield and dry matter content as affected by cropping season and shading degree.

Experimental Treatment	Cycle Length (Days from Transplant)	Yield (t·ha^−1^)	Number of Leaves per Rosette	Mean Weight (g)	Total Dry Matter (g·m^−2^)
Cropping season					
April–May	27.0 ± 3.6 ^b^	10.43 ± 1.61 ^a^	101.2 ± 16.2 ^a^	0.72 ± 0.06 ^b^	78.3 ± 13.3 ^a^
May–June	31.7 ± 3.1 ^a^	9.99 ± 3.54 ^a^	79.9 ± 17.8 ^b^	0.86 ± 0.15 ^a^	85.3 ± 34.2 ^a^
June–July	25.0 ± 2.6 ^b,c^	7.65 ± 1.65 ^b^	88.5 ± 16.8 ^b^	0.60 ± 0.10 ^c^	61.6 ± 16.6 ^b^
July	24.0 ± 3.6 ^c^	4.29 ± 1.19 ^c^	60.6 ± 15.4 ^c^	0.50 ± 0.08 d	46.7 ± 7.8 ^c^
Shading degree (%)					
Unshaded control	24.3 ± 3.9 ^b^	12.16 ± 3.41 ^a^	106.1 ± 14.3 ^a^	0.80 ± 0.24 ^a^	104.1 ± 29.0 ^a^
50	26.0 ± 3.6 ^b^	8.42 ± 1.21 ^b^	84.0 ± 7.5 ^b^	0.71 ± 0.11 ^b^	61.6 ± 9.7 ^b^
79	30.5 ± 3.3 a	6.31 ± 1.49 ^c^	72.7 ± 17.5 ^c^	0.62 ± 0.11 ^c^	42.2 ± 11.4 ^c^

Within each column, means followed by different letters are significantly different according to the Duncan test at *p* ≤ 0.05.

**Table 2:** Colorimetric parameters as affected by cropping season and shading degree.

Treatment	L*	a*	b*
Cropping Season			
April–May	41.1 ± 1.0 ^b^	–14.5 ± 0.9	23.1 ± 1.3
May–June	41.1 ± 1.5 ^b^	–15.3 ± 1.2	21.7 ± 1.4
June–July	42.9 ± 0.5 ^a^	–14.6 ± 0.5	22.6 ± 2.1
July	43.4 ± 0.5 ^a^	–15.1 ± 0.9	21.8 ± 1.1
		n.s.	n.s.
Shading degree (%)			
Unshaded control	42.6 ± 1.6	–14.8 ± 1.1	22.0 ± 2.4
50	42.2 ± 2.3	–14.6 ± 1.0	22.6 ± 1.3
79	41.6 ± 1.7	–15.2 ± 1.0	22.7 ± 1.5
	n.s.	n.s.	n.s.

L*: lightness, from black to white (0 to 100); a* and b*: chroma components (–60 to +60) from green to red and from blue to yellow, respectively. Within each column, n.s.: no statistically significant difference; means followed by different letters are significantly different according to the Duncan test at *p* ≤ 0.05.

**Table 3:** Macroelement content in perennial wall rocket leaves as affected by cropping season and shading degree.

Treatment	NO_3_	N	K	P	S	Ca	Mg	Na
	mg·kg^−1^ f.w.	g·kg^−1^ d.w.	g·kg^−1^ d.w.	g·kg^−1^ d.w.	g·kg^−1^ d.w.	g·kg^−1^ d.w.	g·kg^−1^ d.w.	g·kg^−1^ d.w.
Cropping season								
April–May	6863 ± 488 ^a^	4.50 ± 0.36	47.6 ± 6.3 ^b^	3.02 ± 0.31 ^a^	7.37 ± 0.36	29.3 ± 1.4 ^a^	3.56 ± 0.15 ^a^	3.15 ± 0.27
June–July	6615 ± 303 ^a,b^	4.56 ± 0.41	50.3 ± 4.8 ^a,b^	2.84 ± 0.25 ^a,b^	7.25 ± 0.33	27.8 ± 1.5 ^a,b^	3.37 ± 0.12 ^a,b^	3.24 ± 0.11
July	6404 ± 305 ^b^	4.65 ± 0.43	53.4 ± 3.1 ^a^	2.71 ± 0.23 ^b^	7.18 ± 0.28	25.5 ± 3.0 ^b^	3.14 ± 0.32 ^b^	3.30 ± 0.18
		n.s.			n.s.			n.s.
Shading degree (%)								
Unshaded control	6218 ± 182 ^b^	4.70 ± 0.35	55.5 ± 1.6 ^a^	3.08 ± 0.14 ^a^	7.34 ± 0.33	29.2 ± 1.9 ^a^	3.50 ± 0.20 ^a^	3.30 ± 0.19
50	6594 ± 158 ^b^	4.58 ± 0.44	51.6 ± 2.0 ^b^	2.85 ± 0.11 ^a,b^	7.28 ± 0.23	27.7 ± 1.0 ^a,b^	3.39 ± 0.10 ^a,b^	3.22 ± 0.11
79	7070 ± 336 ^a^	4.44 ± 0.20	44.0 ± 4.7 ^c^	2.65 ± 0.07 ^b^	7.20 ± 0.36	25.5 ± 3.9 ^b^	3.20 ± 0.42 ^b^	3.18 ± 0.42
		n.s.			n.s.			n.s.

f.w.: fresh weight; d.w.: dry weight; n.s.: not statistically significant; Values followed by different letters are statistically different according to the Duncan test at *p* ≤ 0.05.

**Table 4:** Microelement content in perennial wall rocket leaves as affected by cropping season and shading degree.

Treatment	Cu	Fe	Mn	Se	Zn
	mg·kg^−1^ d.w.	mg·kg^−1^ d.w.	mg·kg^−1^ d.w.	µg·kg^−1^ d.w.	mg·kg^−1^ d.w.
Cropping season					
April–May	18 ± 3 ^b^	486 ± 49 ^b^	53 ± 4	237 ± 50 ^c^	45 ± 2 ^a^
June–July	20 ± 2 ^a,b^	515 ± 23 ^a,b^	56 ± 2	272 ± 51 ^b^	42 ± 2 ^a,b^
July	21 ± 3 ^a^	532 ± 58 ^a^	58 ± 7	326 ± 81 ^a^	38 ± 4 ^b^
			n.s.		
Shading degree (%)					
Unshaded control	22 ± 2 ^a^	544 ± 47 ^a^	60 ± 5 ^a^	218 ± 24 ^c^	44 ± 3 ^a^
50	20 ± 1 ^a^	517 ± 22 ^a,b^	56 ± 3 ^a,b^	350 ± 58 ^a^	42 ± 1 ^a,b^
79	17 ± 1 ^b^	472 ± 44 ^b^	51 ± 4 ^b^	267 ± 57 ^b^	39 ± 5 ^b^

d.w.: dry weight; n.s.: not statistically significant. Values followed by different letters are statistically different according to the Duncan test at *p* ≤ 0.05.

**Table 5:** Effect of the interaction between cropping season and shading degree on antioxidant compounds and activity of perennial wall rocket leaves.

Treatment	LAA	HAA	Total Phenols	TAA
	(mmol Trolox 100 g^−1^ d.w.)	(mmol AA 100 g^−1^ d.w.)	(mg gallic acid 100 g^−1^ d.w.)	(mg 100 g^−1^ f.w.)
Cropping season (CS)				
April–May	14.76 ± 0.72 ^c^	7.28 ± 0.89 ^a^	2.45 ± 0.14	79.60 ± 12.0 ^a^
June–July	18.42 ± 1.38 ^b^	6.32 ± 0.75 ^b^	2.52 ± 0.13	65.97 ± 6.72 ^b^
July	20.14 ± 0.75 ^a^	6.71 ± 0.60 ^b^	2.30 ± 0.11	82.08 ± 16.0 ^a^
	*	*	n.s.	*
Shading degree (SD, %)				
Unshaded control	14.84 ± 0.76 ^c^	8.83 ± 0.19 ^a^	2.42 ± 0.15 ^a,b^	119.7 ± 8.30 ^a^
50	17.87 ± 1.19 ^b^	7.41 ± 0.48 ^b^	2.23 ± 0.07 ^b^	57.12 ± 4.55 ^b^
79	20.61 ± 0.88 ^a^	4.08 ± 0.13 ^c^	2.62 ± 0.12 ^a^	50.81 ± 3.37 ^b^
SD × CS				
Control × April–May	13.32 ± 0.33 ^c^	8.98 ± 0.54 ^a^	2.85 ± 0.11 ^a,b^	123.9 ± 2.77 ^b^
Control × June–July	13.71 ± 1.06 ^c^	9.02 ± 0.21 ^a^	2.53 ± 0.14 ^a,b,c^	90.12 ± 4.23 ^c^
Control × July	17.51 ± 0.53 ^b^	8.47 ± 0.11 ^a^	1.88 ± 0.02 ^d^	145.1 ± 5.56 ^a^
50 × April–May	13.61 ± 0.72 ^c^	9.06 ± 0.22 ^a^	2.12 ± 0.14 ^c,d^	72.13 ± 5.72 ^d^
50 × June–July	18.66 ± 0.57 ^b^	6.03 ± 0.41 ^c^	2.12 ± 0.10 ^c,d^	47.21 ± 3.97 ^e,f^
50 × July	21.33 ± 0.96 ^a^	7.13 ± 0.40 ^b^	2.44 ± 0.02 ^b,c^	52.03 ± 4.99 ^e,f^
79 × April–May	17.36 ± 0.65 ^b^	3.81 ± 0.02 ^d^	2.38 ± 0.26 ^c^	42.77 ± 3.61 ^f^
79 × June–July	22.90 ± 0.48 ^a^	3.93 ± 0.16 ^d^	2.90 ± 0.16 ^a^	60.60 ± 5.11 ^d,e^
79 × July	21.57 ± 0.60 ^a^	4.51 ± 0.22 ^d^	2.58 ± 0.13 ^a,b,c^	49.06 ± 3.92 ^e,f^

LAA: lipophilic antioxidant activity; HAA: hydrophilic antioxidant activity; TAA: total ascorbic acid; d.w.: dry weight; f.w.: fresh weight; n.s.: no statistically significant difference; * statistically significant at *p* ≤ 0.05. Within each column, means followed by different letters are significantly different according to the Duncan test at *p* ≤ 0.05. Mean values ± standard deviations have been reported.
